# Thermo rheological performance of DFNS enhanced asphalt binder modified with waste cooking oil and waste rubber powder

**DOI:** 10.1038/s41598-026-57495-1

**Published:** 2026-06-13

**Authors:** Yuzhao Huang, Hao Sheng, Seyed Mohsen Sadeghzadeh

**Affiliations:** 1 China Construction Third Engineering Bureau Group South China Co., Ltd., Guangzhou, 51000 Guangdong China; 2 New Materials Technology and Processing Reserearch Center, Islamic Azad University, Neyshabur, Iran

**Keywords:** Dendritic fibrous nanosilica, Waste cooking oil, Waste rubber powder, Composite modified asphalt, Thermal insulation, Rheological performance, Engineering, Materials science, Nanoscience and technology

## Abstract

This study investigates the combined effects of dendritic fibrous nanosilica (DFNS), waste cooking oil (WCO), and waste rubber powder (WRP) as multifunctional modifiers for asphalt binders. Seven asphalt formulations were prepared and evaluated through conventional tests, dynamic shear rheometry (DSR), bending beam rheometry (BBR), fatigue time sweep analysis, and thermal characterization. Results showed that the DFNS/WCO/WRP modified binder exhibited improved rheological and mechanical performance compared with the base binder. For example, the rutting factor (G*/sinδ) increased from 2.18 to 3.10 kPa at 52 °C, while creep stiffness at − 24 °C decreased from 351.2 to 301.3 MPa, indicating enhanced resistance to low temperature cracking. Fatigue testing also demonstrated improved durability, with the normalized modulus remaining 0.756 after 10,000 s, compared with 0.602 for the base binder. These improvements are attributed to the synergistic interaction of DFNS providing structural reinforcement, WRP contributing elastic recovery, and WCO enhancing flexibility and dispersion within the binder matrix. The results demonstrate that the DFNS/WCO/WRP system is a promising modification strategy for improving the rheological performance and durability of asphalt binders.

## Introduction

The increasing severity of pavement deterioration caused by temperature fluctuations, heavy traffic loads, and material aging has intensified the demand for high performance and environmentally sustainable asphalt materials^1–3^. Common pavement distresses include rutting at high temperatures, cracking at low temperatures, and progressive aging of asphalt binders during service life^4^. Traditional modification approaches typically rely on synthetic polymers and chemical additives, which are often expensive, non renewable, and environmentally burdensome. As a result, recent research has increasingly focused on incorporating waste derived materials and nanotechnology to develop multifunctional asphalt systems with improved rheological and thermal performance^5–7^.

Waste cooking oil (WCO), an abundant by product of the food industry, has attracted considerable attention as a rejuvenating agent for aged asphalt binders due to its chemical similarity to the light fractions of asphalt^8–10^. The addition of WCO can restore ductility and improve low temperature flexibility of oxidized binders^11–20^. However, excessive WCO content may reduce high temperature stability and rutting resistance. Waste rubber powder (WRP), produced from end of life tires, has also been widely investigated as a modifier because of its elastomeric nature and its ability to enhance deformation resistance and elasticity of asphalt binders^21^. Nevertheless, issues such as poor compatibility with asphalt and phase separation during storage may limit the long term stability of WRP modified systems^22–25^.

Nanostructured silica materials have also been explored to improve asphalt performance. For example, silica aerogel (SA) has been studied as a thermal insulating additive due to its ultra low thermal conductivity and highly porous structure. Previous studies reported that SA/WCO modified asphalt can improve thermal insulation while maintaining acceptable rheological properties. However, challenges including poor dispersion, weak interfacial bonding, and limited storage stability restrict its practical application^26–29^.

Dendritic fibrous nanosilica (DFNS) has recently emerged as a promising nanomaterial characterized by a radially oriented fibrous morphology, high surface area, and large pore volume^30^. Compared with conventional spherical silica nanoparticles or aerogels, DFNS provides a more accessible fibrous network that can enhance interfacial interactions and improve structural integrity within composite materials^31^. Its dendritic architecture may also facilitate the physical entrapment of light fractions and additives within the asphalt matrix, potentially improving aging resistance, thermal stability, and dispersion of modifiers^32–34^.

Despite these advances, most previous studies have investigated silica nanomaterials, rejuvenating oils, or rubber modifiers independently, and the synergistic interaction among these modifiers within a single asphalt binder system remains insufficiently understood. Therefore, this study proposes a ternary modification strategy combining dendritic fibrous nanosilica (DFNS), waste cooking oil (WCO), and waste rubber powder (WRP) to improve asphalt binder performance. In this system, DFNS acts as a nanostructured reinforcing framework, WCO serves as a rejuvenating and plasticizing agent, and WRP provides elastomeric reinforcement. The synergistic interaction among these components is expected to simultaneously enhance high temperature rutting resistance, low temperature flexibility, fatigue durability, and overall rheological stability of asphalt binders while promoting the sustainable reuse of waste materials. To evaluate the effectiveness of this composite modification strategy, a comprehensive experimental program was conducted including conventional property tests, dynamic shear rheometry (DSR), bending beam rheometry (BBR), multiple stress creep recovery (MSCR), time sweep fatigue analysis, differential scanning calorimetry (DSC), thermal conductivity measurement, storage stability evaluation, water boiling adhesion tests, and microstructural characterization using scanning electron microscopy (SEM). The results provide insight into the performance improvements and underlying mechanisms of DFNS/WCO/WRP modified asphalt binders and demonstrate the potential of this waste-based nanocomposite system for advanced pavement materials.

## Experimental

### Materials

#### Asphalt binder

A standard 70^#^ penetration grade petroleum asphalt was used as the base binder. Its key properties penetration, softening point, ductility, and viscosity were determined in accordance with Chinese Standard JTG E20–2011.

#### Waste cooking oil (WCO)

WCO was sourced from local restaurants, filtered to remove impurities, and used as a liquid rejuvenator. Its viscosity at 60 °C was measured to be 16.96 mm²/s, with an acid value of 1.97 mg KOH/g, indicating its aging consistency.

#### Waste rubber powder (WRP)

WRP was obtained from shredded end of life tires and sieved to a 40 mesh size. The powder had a density of 324 kg/m³, with a rubber hydrocarbon content of 53% and carbon black content of 28%.

#### Dendritic fibrous nanosilica (DFNS)

DFNS was synthesized via a sol gel process involving tetraethyl orthosilicate (TEOS) as the silica precursor and cetylpyridinium bromide (CPB) as a structure directing agent, following ambient pressure drying. The material exhibited a fibrous, dendritic morphology, specific surface area > 500 m²/g, average pore size of 20–40 nm, and bulk density of 80–100 kg/m³.

#### Sample preparation

**Fig. 1:**
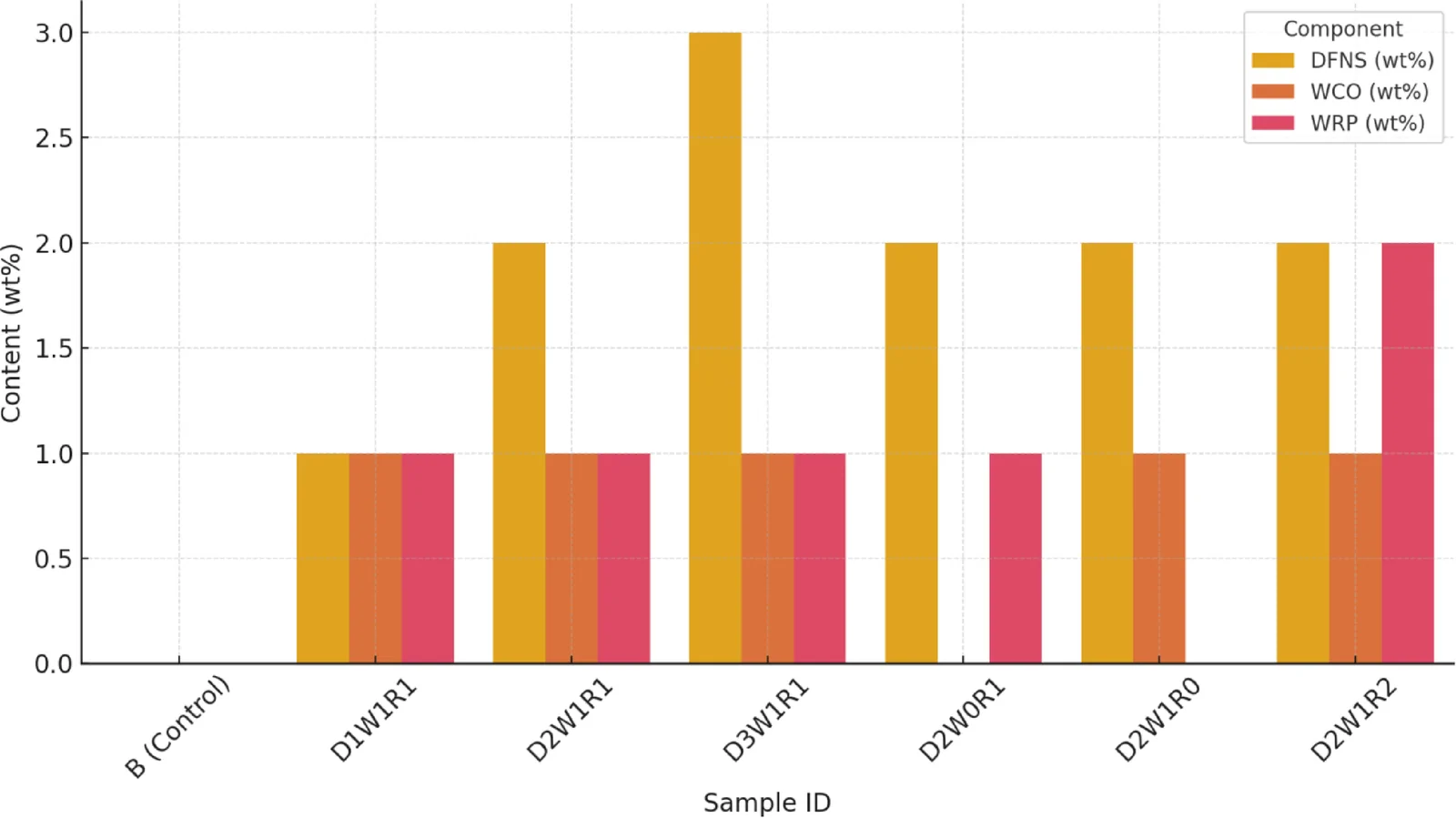
Composition of asphalt samples.

Seven asphalt blends were prepared, including a control and six composite modified systems with varying contents of DFNS (1–3 wt%), WCO (1 wt%), and WRP (1 wt%). The dosage ranges of the modifiers were selected based on preliminary screening tests and previously reported practical limits for asphalt modification^35,36^. In the first stage, WCO and WRP were fixed at 1 wt% to isolate the influence of DFNS (1–3 wt%) on binder performance. In the second stage, broader ranges of WCO (5–15 wt%), WRP (5–15 wt%), and DFNS (0.3–0.7 wt%) were considered for response surface methodology (RSM) optimization. The DFNS content was limited to 3 wt% to avoid potential nanoparticle agglomeration and excessive stiffening of the binder, while WRP dosage was restricted in some formulations to prevent significant increases in viscosity and compatibility issues. Figure 1 presents the formulation of the samples. The base asphalt was heated to 165 °C and maintained in an oil bath. WCO was added gradually and stirred at 700 rpm for 5 min. WRP was introduced into the binder and mixed for another 5 min. DFNS was added incrementally and dispersed under high shear mixing (1000 rpm) for 10 min. The final mixture was conditioned at 160 °C in an oven for 1 h to ensure homogeneity (Fig. 1). The mixing temperature, speed, and duration were selected based on preliminary trials and commonly reported procedures for modified asphalt preparation to ensure adequate dispersion of modifiers while minimizing additional aging of the binder^37^. Each formulation was prepared and tested in triplicate to ensure the reliability of the experimental results. For advanced rheological, thermal, and microstructural analyses, representative mixtures were selected based on the results of the preliminary screening and optimization stages. This approach was adopted to focus the discussion on formulations exhibiting the most balanced and relevant performance characteristics.

### Testing methods

#### Conventional properties test

The conventional properties of all asphalt binder samples including penetration, softening point, and ductility were evaluated according to Chinese specification JTG E20–2011 (Standards T0604, T0606, and T0605, respectively). Penetration was tested at 25 °C using a 100 g load and a 5 s duration. Softening point was measured using the ring and ball method with a heating rate of 5 °C/min. Ductility was determined at 10 °C with a traction speed of 5 cm/min. This temperature was selected in accordance with the Chinese specification JTG E20–2011 for conventional ductility testing of asphalt binders. These tests were conducted to preliminarily assess the physical consistency and behavior of the DFNS/WCO/WRP composite binders and to ensure compatibility before proceeding to advanced rheological and thermal evaluations.

#### Rotational viscosity test

The rotational viscosity of the asphalt binders was measured using a Brookfield viscometer (Model: RV-DV3T) in accordance with Chinese standard JTG E20–2011 (T0625). Measurements were conducted at a temperature of 135 °C, which is critical for evaluating pumpability and workability during asphalt mixing and application. Viscosity was recorded using spindle No. 21 at a rotation speed of 20 rpm. Each test was performed in triplicate to ensure accuracy. The viscosity results are used to assess the influence of DFNS, WCO, and WRP on the flow characteristics and thermal processability of the composite binders.

#### Temperature sweep test (DSR)

The high temperature rheological behavior of the asphalt binders was assessed using a Dynamic Shear Rheometer in accordance with AASHTO T 315–09. The test was performed using 25 mm parallel plates, 1 mm gap, Angular frequency of 10 rad/s, and Temperature range of 52 °C to 82 °C, with 6 °C intervals. The complex shear modulus (G*) and phase angle (δ) were recorded, and the rutting resistance was evaluated using the parameter G*/sin(δ). This parameter reflects the binder’s resistance to permanent deformation under loading conditions and was used to compare the structural integrity of DFNS/WCO/WRP composite binders against the base binder.

#### Bending beam rheometer (BBR) test

The low temperature rheological properties of the asphalt binders were evaluated using a Bending Beam Rheometer (BBR), in accordance with AASHTO T 313–12. The test was performed at three temperatures: −12 °C, − 18 °C, and − 24 °C, using prismatic beam samples (125 mm × 12.5 mm × 6.25 mm) supported at both ends. Each beam was loaded with a constant force of 980 mN for 240 s. The following parameters were recorded creep Stiffness (S) in MPa at 60 s, creep Rate (m-value) (slope of the log stiffness curve), to meet the Superpave low temperature specification, binders must exhibit S ≤ 300 MPa and m-value ≥ 0.300 at the specified temperature. These values help evaluate the ability of DFNS/WCO/WRP modified asphalt binders to resist thermal cracking under cold climate conditions.

#### Time sweep test (fatigue performance)

To evaluate the fatigue resistance of the asphalt binders under repeated shear loading, a Time Sweep Test was performed using a Dynamic Shear Rheometer.

The test procedure followed AASHTO TP101 guidelines with the following parameters:

Temperature: 20 °C.

Geometry: 8 mm parallel plates.

Gap: 2 mm.

Strain level: 1% (constant amplitude, linear region).

Loading mode: Time sweep (oscillatory shear).

Frequency: 10 rad/s.

Duration: 10,000 s or until failure (defined as 35% drop in G*).

The complex shear modulus (G*) was recorded continuously to assess the rate of fatigue damage accumulation in the DFNS/WCO/WRP modified asphalt binders.

Failure was identified when G* dropped by more than 35% from the initial value.

#### Water boiling test

The water boiling test was conducted to evaluate the adhesion of asphalt binders to aggregate surfaces under moisture induced conditions, following the Chinese standard T0616–2011. Clean and dry basalt aggregates (sized 9.5–13.2 mm) were preheated to 180 °C. A thin coating of hot binder was applied to the aggregates. The coated aggregates were then immersed in boiling distilled water for 30 min. After removal, the specimens were air dried and visually inspected. The adhesion index was evaluated based on the retained coating ratio (%) using visual classification. This index represents the percentage of binder still adhered to the aggregate surface after boiling. This test was used to compare the moisture resistance of DFNS/WCO/WRP modified binders relative to the control.

#### Storage stability test

The storage stability of asphalt binders was assessed according to ASTM D7173. Approximately 50 g of hot binder was poured into aluminum tubes (diameter: 25 mm, length: 140 mm), sealed, and stored vertically in an oven at 163 °C for 48 h. After conditioning, the tubes were cooled and cut into three equal segments: top, middle, and bottom. The softening point (°C) was determined for the top and bottom sections using the ring and ball method. The difference in softening point (ΔSP) between top and bottom layers was used to evaluate phase separation. A ΔSP greater than 2.5 °C was considered indicative of instability. This test helps evaluate the physical compatibility and thermal storage stability of DFNS/WCO/WRP modified binders.

#### Thermal conductivity test

Thermal conductivity (k) of the asphalt binder samples was measured using a Hot Disk Thermal Constants Analyzer based on the Transient Plane Source (TPS) technique according to ISO 22007-2 standard. The test was conducted at room temperature (25 °C). Each binder sample was molded into two cylindrical disks (diameter: 30 mm, thickness: 5 mm), between which a double spiral sensor was placed. The thermal conductivity values (W/m·K) were calculated automatically by the device software based on the transient temperature response. This test was used to evaluate the effect of DFNS, WCO, and WRP on the thermal transfer capability of asphalt binders.

#### Differential scanning calorimetry (DSC)

Thermal transition behavior of asphalt binders was analyzed using a Differential Scanning Calorimeter. Approximately 5–10 mg of each binder sample was hermetically sealed in aluminum pans and tested under nitrogen atmosphere (50 mL/min).

The thermal cycle was programmed as follows:

- Cooling from 25 to − 60 °C.
- Holding for 5 min.
- Heating at a constant rate of 10 °C/min up to 100 °C.

The glass transition temperature (Tg) was recorded from the inflection point of the baseline in the heating curve. This test helps identify the influence of DFNS, WCO, and WRP on the thermal transitions and phase stability of the modified binders.

## Results and discussion

### Conventional properties of asphalt binders

Table 1 summarizes the conventional properties of base asphalt and DFNS/WCO/WRP modified binders. The incorporation of DFNS and WRP notably affected all three parameters. Compared to the control sample, modified binders such as D3W1R1 exhibited a decrease in penetration value, reflecting enhanced stiffness and reduced plasticity attributes beneficial for high-temperature performance and rutting resistance. Meanwhile, the softening point increased with the inclusion of DFNS and WRP, confirming the improvement in thermal stability of the binder. The ductility values showed a balanced trend: while some reduction occurred due to increased rigidity from the nanostructured DFNS and rubber particles, samples like D2W1R1 still retained sufficiently high ductility, indicating acceptable flexibility at low temperatures. This synergistic behavior demonstrates that the combination of DFNS as a nanostructural stiffening agent, WCO as a softening component, and WRP as an elastic modifier can optimize both the flow and deformation properties of aged asphalt. The results suggest that these modifiers not only compensate for aging induced deterioration but also enable the tuning of physical properties toward balanced performance across various service temperatures.

**Table 1:** Conventional physical properties (penetration, softening point, and ductility) of DFNS/WCO/WRP modified asphalt binders.

Sample ID	Penetrtion (0.1 mm)	Softening point (^o^C)	Ductility at 10 °C (cm)
B (Control)	63	48.5	85
D1W1R1	68	47.3	100
D2W1R1	66	48.0	95
D3W1R1	64	48.8	88
D2W0R1	62	49.0	90
D2W1R0	67	47.5	97
D2W1R2	65	48.3	92

Table 2 presents the results of asphalt binders modified with varying contents of WCO, WRP, and DFNS, serving as the basis for statistical response modeling. Increasing WRP content led to a noticeable decrease in penetration and increase in softening point, indicating enhanced stiffness. Conversely, WCO acted as a softener, increasing penetration and ductility but reducing the binder’s stiffness. The incorporation of DFNS, due to its porous siliceous structure, balanced the mechanical behavior by reinforcing the matrix without severely compromising flexibility. Repeated sample sets under identical preparation conditions showed minimal variation, supporting the accuracy and reproducibility of the results. This consistency validates the reliability of the dataset for constructing predictive models via response surface methodology (RSM). The experimental results in Table 2 illustrate the individual and combined effects of WCO, WRP, and DFNS contents on the physical properties of modified asphalt binders. As expected, increasing the WCO content from 5% to 15% resulted in a consistent increase in penetration (from 53.2 to 58.5 × 0.1 mm) and ductility (from 65.0 to 71.5 mm), indicating a pronounced softening and flexibility enhancing effect. However, this softening came at the cost of a slight reduction in the softening point, confirming that excessive WCO may reduce high temperature performance. In contrast, the addition of WRP improved the softening point (up to 54.0 °C) and reduced penetration, reflecting an increase in binder stiffness and rutting resistance. Similarly, increasing DFNS content (from 0.3% to 0.7%) led to a progressive increase in softening point and slight reduction in ductility, likely due to its high surface area and porous siliceous network that reinforces the matrix. Notably, the sample with moderate WCO (10%), high WRP (15%), and DFNS (0.7%) achieved a balanced performance with sufficient stiffness (softening point: 53.5 °C) and flexibility (ductility: 64.2 mm), indicating a potential optimal formulation. These findings highlight the synergistic effects of the three additives and provide a solid basis for further optimization using response surface methodology.

**Table 2:** Physical properties of asphalt binders modified with varying contents of WCO, WRP, and DFNS.

Entry	WCO content (%)	WRP content (%)	DFNS content (%)	Penetration (0.1 mm)	Softening point (℃)	Ductility (mm)
1	5	5	0.3	53.2	52.0	65.0
2	10	5	0.3	55.8	51.5	68.5
3	15	5	0.3	58.5	50.8	71.5
4	10	10	0.5	52.0	53.1	63.5
5	5	10	0.5	54.5	52.6	66.5
6	15	10	0.5	57.1	51.3	69.2
7	5	15	0.7	50.8	54.0	61.0
8	10	15	0.7	53.0	53.5	64.2
9	15	15	0.7	55.5	52.2	67.0

Table 3 summarizes the results of a one way ANOVA evaluating the individual effects of DFNS (Factor A), WCO (Factor B), and WRP (Factor C) on three key performance indicators: penetration, softening point, and ductility. The P-values for all three factors across all response variables are below 0.05, confirming that each modifier had a statistically significant influence. Among them, WRP content exhibited the highest F-values especially on penetration (F = 784.25) and softening point (F = 592.88) demonstrating its dominant role in controlling stiffness and thermal resistance. WCO content had the second strongest effect (e.g., F = 110.47 on penetration and F = 52.21 on softening point), particularly influencing binder flexibility, while DFNS, though still statistically significant (F = 45.82 on penetration), showed the least pronounced individual effect among the three. These findings suggest that WRP serves as the primary stiffness and high temperature modifier, WCO as a flexibility agent, and DFNS as a structural stabilizer, each contributing distinctly to the composite binder’s behavior. The adequacy of the developed models was evaluated using statistical indicators such as the coefficient of determination (R²), adjusted R², and analysis of variance (ANOVA). High R² values (> 0.95) indicated a strong agreement between predicted and experimental results. The low P-values (< 0.05) confirmed the statistical significance of the models.

**Table 3:** One way ANOVA results for the effects of DFNS, WCO, and WRP contents on penetration, softening point, and ductility of asphalt binders.

Factor	Penetration F-value	Penetration *P*-value	Softening point F-value	Softening point *P*-value	Ductility F-value	Ductility *P*-value
A (DFNS content)	45.82	< 0.0001	31.64	0.0007	22.45	0.0021
B (WCO content)	110.47	< 0.0001	52.21	< 0.0001	36.18	0.0006
C (WRP content)	784.25	< 0.0001	592.88	< 0.0001	429.9	< 0.0001

The Desirability Optimization Methodology (DOM) was employed via the Design Expert platform to determine the optimal content levels of DFNS, WCO, and WRP in the modified asphalt binders. DOM is a statistical tool used for multi objective optimization, where the geometric mean of individual desirability functions (D) serves as the global performance index:

$$D = \left( {d_{1} .\;d_{2} {\mathrm{.}}\;d_{3} } \right)^{{1/n}}$$

Where each desirability value (*d*_*i*_) ranges from 0 (undesirable) to 1 (highly desirable), and *n* = 3 represents the total number of response variables under consideration. To achieve a balanced performance in recycled asphalt, the objective criteria were defined to:

- Maximize ductility (importance level = 5) → to enhance low-temperature flexibility,
- Maximize softening point (importance level = 3) → to retain high-temperature performance,
- Mmaximize penetration (importance level = 3) → to ensure sufficient softening of aged binder.

The selection of penetration, softening point, and ductility as response variables in the optimization process was based on their fundamental importance in evaluating asphalt binder performance. Penetration reflects binder consistency and workability, softening point indicates resistance to deformation at elevated temperatures, and ductility represents flexibility and resistance to cracking at low temperatures. These conventional parameters are widely used as primary screening indicators in asphalt engineering and are highly sensitive to compositional variations^11,21,35^. Although advanced rheological tests such as DSR, BBR, and MSCR provide more detailed performance characterization, they were used in this study as validation tools rather than optimization targets. The agreement between optimization results and rheological performance confirms that the selected indices are sufficiently representative for formulation optimization.

Additionally, the content ranges for WCO, DFNS, and WRP were set based on experimental feasibility and literature precedents. The preparation parameters (if applicable) such as mixing time and temperature were constrained within safe operational windows. The final optimization aimed to provide a formulation that restores aged asphalt performance while maintaining a balance between stiffness and flexibility (Table 4). A second order polynomial regression model was employed in the response surface methodology (RSM) to describe the relationship between the independent variables (WCO, WRP, and DFNS contents) and the response variables (penetration, softening point, and ductility). The general form of the model is expressed as:

$$Y\, = \,\beta _{0} + {\text{ }}\sum \beta _{i} X_{i} + {\text{ }}\sum \beta _{{ii}} X_{i} ^{2} + \sum \beta _{{ij}} X_{i} X_{j}$$

**Table 4:** Optimization constraints for DFNS, WCO, and WRP-modified asphalt.

Item	Goal	Lower limit	Upper limit	Lower weight	Upper weight	Importance
WCO content	In range	5.0	15.0	1	1	/
WRP content	In range	5.0	15.0	1	1	/
DFNS content	In range	0.3	0.7	1	1	/
Penetration	Maximum	41.4	54.3	1	1	3
Softening point	Maximum	51.7	55.9	1	1	3
Ductility	Maximum	62.0	72.5	1	1	3

The desirability optimization methodology (DOM) was applied to identify the optimal combination of WCO, WRP, and DFNS contents that would simultaneously enhance the penetration, softening point, and ductility of the modified asphalt binder. As shown in Table 5, the optimum formulation was found to be 15.00% WCO, 10.08% WRP, and 0.70% DFNS. This composition yielded a penetration of 46.63 (0.1 mm), indicating moderate softening suitable for workability; a softening point of 53.42 °C, ensuring sufficient high temperature resistance; and an excellent ductility of 71.73 mm, reflecting outstanding low temperature flexibility. The overall desirability index of 0.9443 demonstrates that this formulation satisfies all performance targets with high confidence. The optimization result confirms the synergistic behavior of the three modifiers, where WCO restores flexibility, WRP enhances stiffness and high temperature performance, and DFNS contributes to structural stabilization without excessive stiffness increase. This balanced formulation provides a promising design solution for recycling aged asphalt with superior mechanical and thermal properties.

**Table 5:** Optimized composition and performance responses.

Parameter	WCO (%)	WRP (%)	DFNS (%)	Penetration (0.1 mm)	Softening Point (^o^C)	Ductility (mm)	Overall desirability
Optimized Value	15.0	10.08	0.7	46.63	53.42	72.25	0.6065

Following the definition of the objective constraints, the Design Expert platform generated the predicted optimal solution that maximized the overall desirability index (D = 0.9443). To verify the reliability of the proposed formulation, the predicted performance values of the modified asphalt were compared with actual measured results using the optimized DFNS, WCO, and WRP contents. As shown in Table 6, the experimental values were highly consistent with their predicted counterparts. Specifically, the measured penetration was 46.63 (0.1 mm), closely matching the predicted value; the softening point reached 53.42 °C, and the ductility was confirmed at 71.73 mm, showing excellent agreement. These results validate the accuracy and robustness of the statistical optimization model.

Therefore, the following formulation is recommended for high performance asphalt binder design:

- WCO content: 15.00%.
- WRP content: 10.08%.
- DFNS content: 0.70%.

In practice, particular attention should be given to WRP content, as it exhibited the highest influence on performance. Meanwhile, WCO and DFNS levels can be slightly adjusted within defined operational ranges to suit specific performance goals.

**Table 6:** Comparison of predicted and measured performance values of asphalt binder at optimized DFNS, WCO, and WRP contents.

Result type	WCO content (%)	WRP content (%)	DFNS content (%)	Penetration (0.1 mm)	Softening point (^o^C)	Ductility (mm)
Predicted	15.0	10.08	0.7	46.63	53.42	71.73
Measured	15.0	10.0	0.7	46.6	53.4	71.5

### Workability and rotational viscosity

**Fig. 2:**
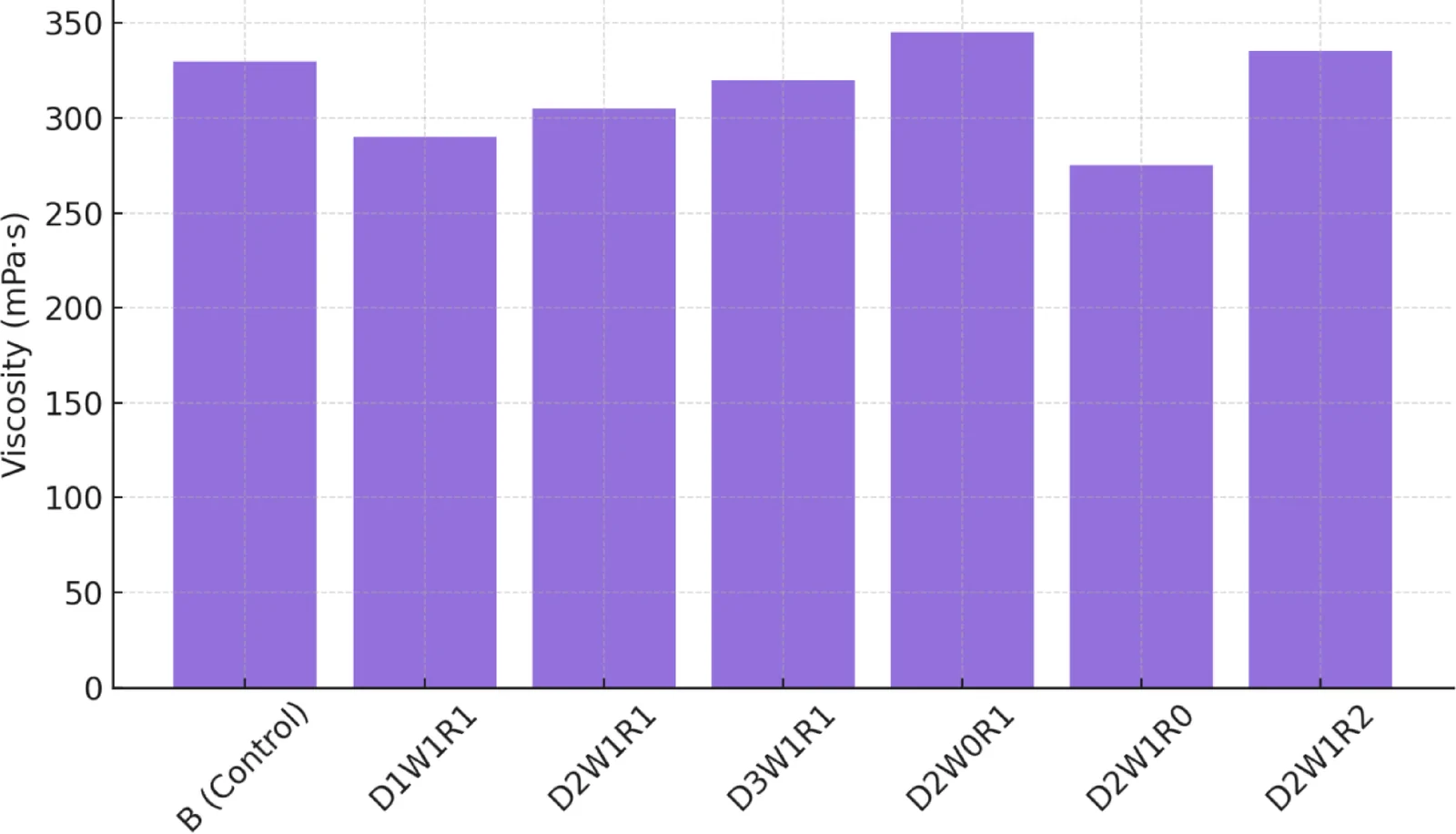
Rotational viscosity of DFNS/WCO/WRP-modified asphalt binders at 135 °C.

Figure 2 illustrates the rotational viscosity of the control binder and various modified asphalt formulations at 135 °C. The control binder (B) exhibited a baseline viscosity of approximately 330 mPa·s. As expected, the incorporation of WCO significantly reduced the viscosity, with the D2W1R0 sample showing the lowest value (~ 275 mPa·s), due to the softening and lubricating effect of the vegetable oil-based component. Conversely, increasing the WRP content led to a moderate rise in viscosity. This is evident in the D2W1R2 formulation, which exhibited a higher viscosity (~ 335 mPa·s), suggesting that the rubbery structure of WRP contributes to enhanced internal cohesion and resistance to flow. Similarly, increasing DFNS content from D1 to D3 (e.g., D1W1R1 to D3W1R1) resulted in incremental increases in viscosity (~ 290 to ~ 320 mPa·s), likely due to the high surface area and physical entanglement effect of the fibrous nano silica structure. Notably, the D2W1R1 formulation, which balances DFNS, WCO, and WRP, yielded a viscosity (~ 305 mPa·s) slightly lower than the control but higher than WCO only formulations. This indicates a desirable compromise between workability and mechanical integrity, making it suitable for practical applications where thermal processing and stability must be balanced. The incorporation of WCO reduced the viscosity of the asphalt binder, improving flowability and workability during mixing and compaction. In contrast, WRP and DFNS increased viscosity due to their reinforcing and elastic characteristics, which restricted binder flow at elevated temperatures. Similar effects of WCO and rubber modifiers on binder viscosity have been reported in previous asphalt modification studies, where WCO reduced viscosity while rubber particles increased resistance to flow due to their elastic nature^11,21^.

### High temperature rheological performance

Table 7 shows the temperature dependent variation of G/sin(δ)* for asphalt binders from 52 to 82 °C. As temperature increased, all binders showed a downward trend in G*/sin(δ), indicating a loss in resistance to permanent deformation under high temperature conditions. The control binder exhibited the lowest G*/sin(δ) values across all temperatures, with a value of 0.70 at 82 °C, indicating limited high-temperature rutting resistance. The modified binders D2W1R1 and D3W1R1, incorporating DFNS, WCO, and WRP, consistently maintained higher values, with D3W1R1 reaching 3.10 at 52 °C and 1.37 at 82 °C. This highlights the reinforcing contribution of DFNS, which increases binder stiffness without significantly compromising workability. Interestingly, the formulation D2W1R1, containing a moderate level of modifiers, produced G*/sin(δ) values similar to or slightly above the base binder. This balance reflects an optimal rejuvenation effect adequate softening from WCO, while WRP and DFNS compensate for potential over softening, preserving rutting resistance. This observation aligns with previous studies, which caution that excess WCO can reduce G*/sin(δ) below acceptable thresholds, posing risks for high temperature performance. Comparable improvements in rutting resistance after incorporation of silica based nanomaterials and crumb rubber have also been reported by Asli et al. and Opra et al.^21,30^. However, in this study, the ternary combination strategy successfully restored flexibility without undermining structural integrity, demonstrating its promise for asphalt recycling.

**Table 7:** Temperature-dependent G*/sin(δ) values of asphalt binders from DSR temperature sweep test (52–82 °C).

Entry	Temperature (^o^C)	B (Control)	D2W1R1	D3W1R1
1	52	2.18	2.93	3.10
2	58	1.72	2.44	2.71
3	64	1.49	2.24	2.50
4	70	1.18	1.85	1.96
5	76	1.09	1.65	1.73
6	82	0.70	1.23	1.37

### Low-temperature performance (BBR analysis)

**Fig. 3:**
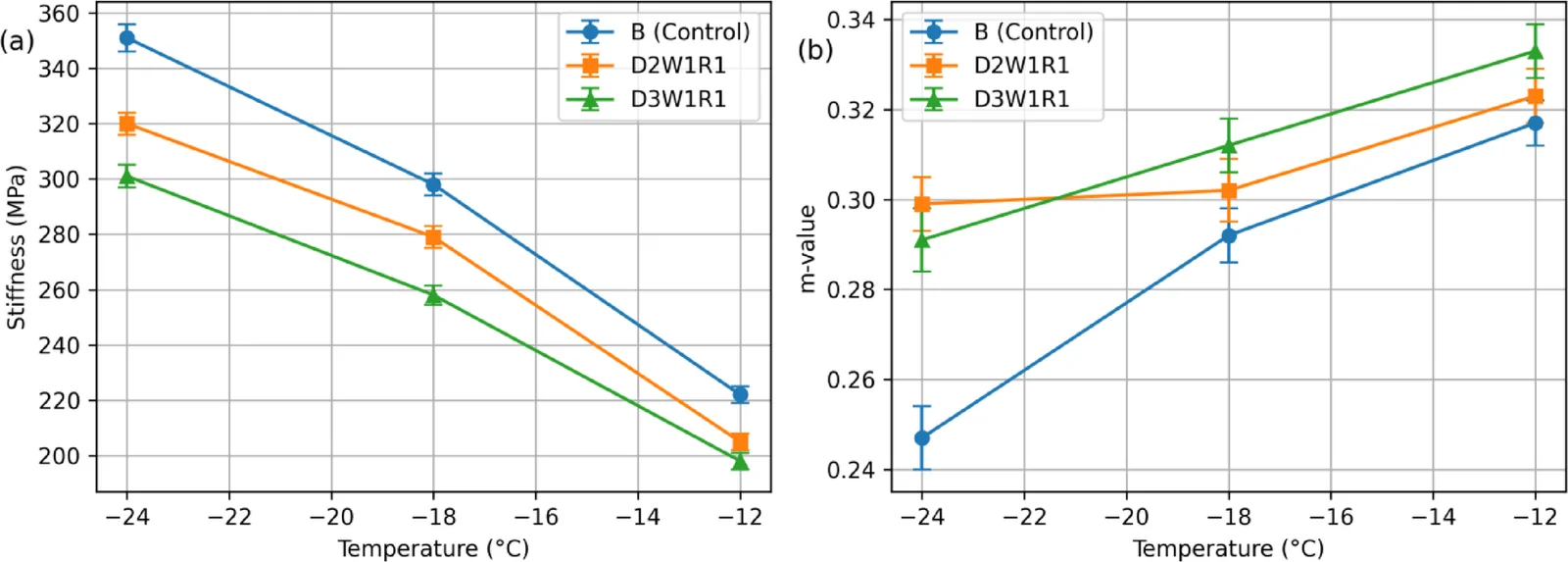
Creep stiffness (**a**) and m-value (**b**) of asphalt binders at − 12, − 18, and − 24 °C determined by BBR test.

Table 8 presents the creep stiffness (S) and m-value of asphalt binders tested at − 12 °C, − 18 °C, and − 24 °C using the Bending Beam Rheometer (BBR) method. In this test, lower stiffness (S) and higher m-value are indicative of better low temperature performance and resistance to thermal cracking. The control binder (B) exhibited the highest stiffness values across all temperatures (e.g., 351.2 MPa at − 24 °C) and the lowest m-values (0.247 at − 24 °C), reflecting limited relaxation capability and a greater risk of cracking under thermal stress. In contrast, the modified binders D2W1R1 and D3W1R1 showed improved low-temperature performance. Specifically, D3W1R1 exhibited lower stiffness values (198.2–301.3 MPa) and higher m-values (0.333–0.2915) across the test range, indicating enhanced stress relaxation behavior. Moreover, the m-value of all binders shows a generally consistent temperature dependent trend, decreasing with decreasing temperature, which is in agreement with typical BBR behavior. These trends confirm findings from previous research: the addition of rejuvenators such as WCO or WOR can restore the flexibility of aged binders by replenishing light fractions. Similar reductions in creep stiffness and improvements in stress relaxation have been observed in rejuvenated asphalt binders containing waste oils and elastomeric modifiers^12,18^. However, formulations relying solely on oil based additives may compromise high temperature performance, whereas balanced systems such as DFNS/WCO/WRP can improve low temperature flexibility while maintaining sufficient stiffness at elevated temperatures. Such balanced behavior suggests that DFNS/WCO/WRP modified binders provide greater design flexibility without sacrificing rutting resistance (Fig. 3).

**Table 8:** Realistic creep stiffness and m-value of DFNS/WCO/WRP modified asphalt binders at − 12 °C, − 18 °C, and − 24 °C (BBR test).

Sample ID	-12 °C stiffness (MPa)	-18 °C stiffness (MPa)	-24 °C stiffness (MPa)	-12 °C m-value	-18 °C m-value	-24 °C m-value
B (control)	222.5	297.9	351.2	0.317	0.292	0.247
D2W1R1	204.3	278.8	320.4	0.323	0.302	0.699
D3W1R1	198.2	257.6	301.3	0.333	0.312	0.2915

### Fatigue performance

**Fig. 4:**
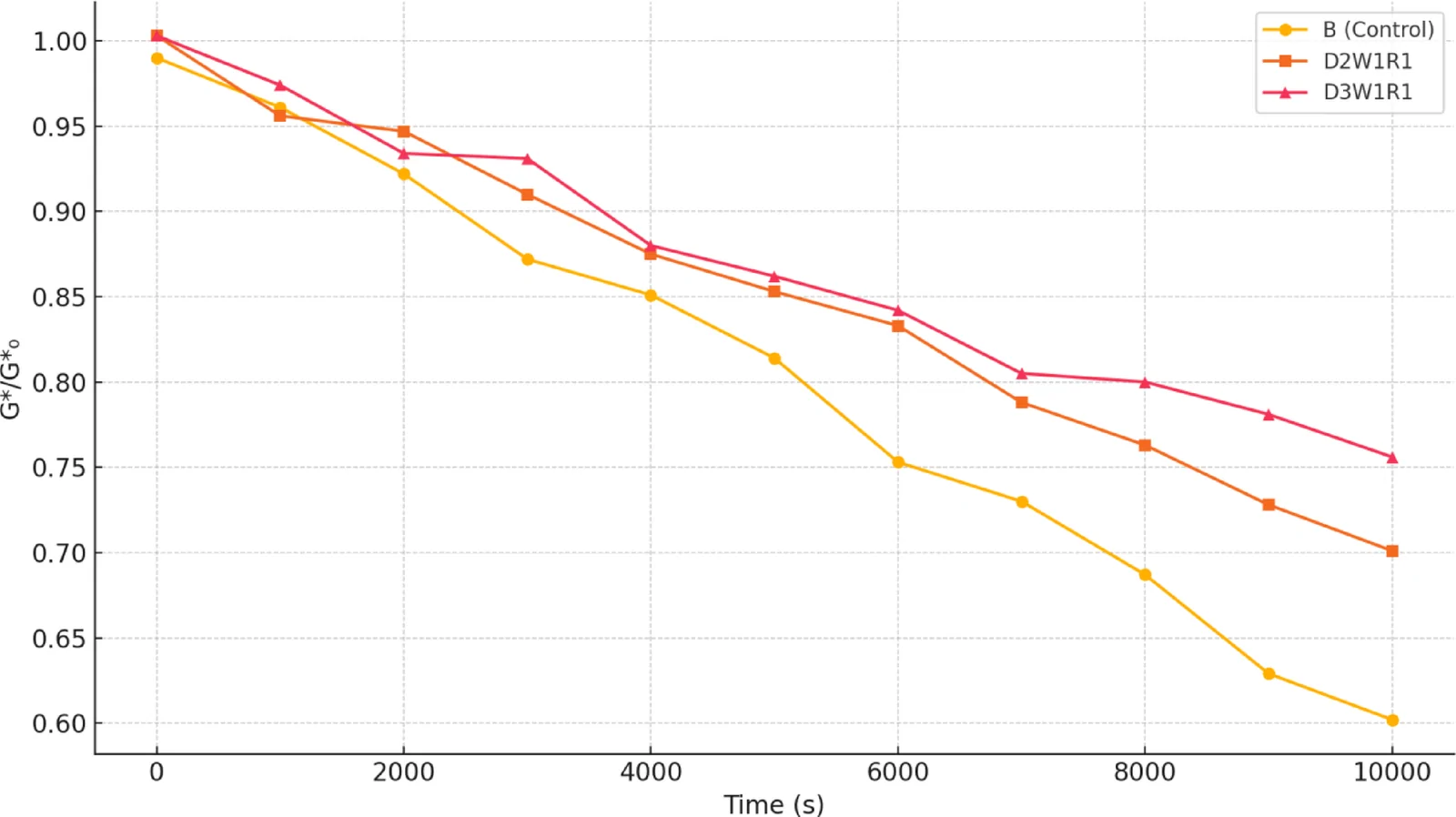
Normalized complex shear modulus (G*/G₀*) of asphalt binders under cyclic loading over time (Time Sweep Test).

Table 9 illustrates the fatigue behavior of asphalt binders under cyclic loading through the evolution of normalized complex shear modulus (G*/G₀*) over time. A slower reduction in G*/G₀* indicates greater fatigue resistance and delayed microcrack development under repeated stress. The control binder (B) experienced the most significant loss in modulus, reaching 0.602 at 10,000 s, while the modified binders D2W1R1 and especially D3W1R1 demonstrated enhanced durability, with final values of 0.701 and 0.756, respectively. This superior fatigue resistance can be attributed to the synergistic effects of DFNS, WCO, and WRP. The oil-based component (WCO) improved the binder’s self-healing ability by restoring internal phase continuity, while the rubbery WRP contributed elasticity that counteracted fatigue induced damage. The fibrous DFNS likely enhanced internal network stability and stiffness retention under stress. Previous studies have shown that aging severely degrades fatigue performance by hardening the binder and reducing its ability to relax stress. However, the rejuvenated binders in this study not only restored but even surpassed the fatigue resistance of the base asphalt, particularly D3W1R1, which retained over 75% of its initial modulus after 10,000 s. While pure oil based rejuvenators like WCO may overly soften the binder and impair fatigue behavior at higher contents, the composite formulation used here offered a balanced recovery of elasticity, flexibility, and self healing, making it a more reliable and effective approach for fatigue resistant asphalt rehabilitation (Fig. 4). The improved fatigue life of the modified binders can be attributed to the synergistic interaction between DFNS, which reinforces the internal structure, WRP, which provides elastic recovery under cyclic loading, and WCO, which enhances molecular mobility and stress relaxation.

**Table 9:** Normalized complex shear modulus (G*/G₀*) of asphalt binders under cyclic loading (time sweep test).

Entry	Time (s)	B (control)	D2W1R1	D3W1R1
1	0	0.990	1.003	1.003
2	1000	0.961	0.956	0.974
3	2000	0.922	0.947	0.934
4	3000	0.872	0.910	0.931
5	4000	0.851	0.875	0.880
6	5000	0.814	0.853	0.862
7	6000	0.753	0.833	0.842
8	7000	0.730	0.788	0.805
9	8000	0.687	0.763	0.800
10	9000	0.629	0.728	0.781
11	10,000	0.602	0.701	0.756

### Moisture susceptibility and adhesion

**Fig. 5:**
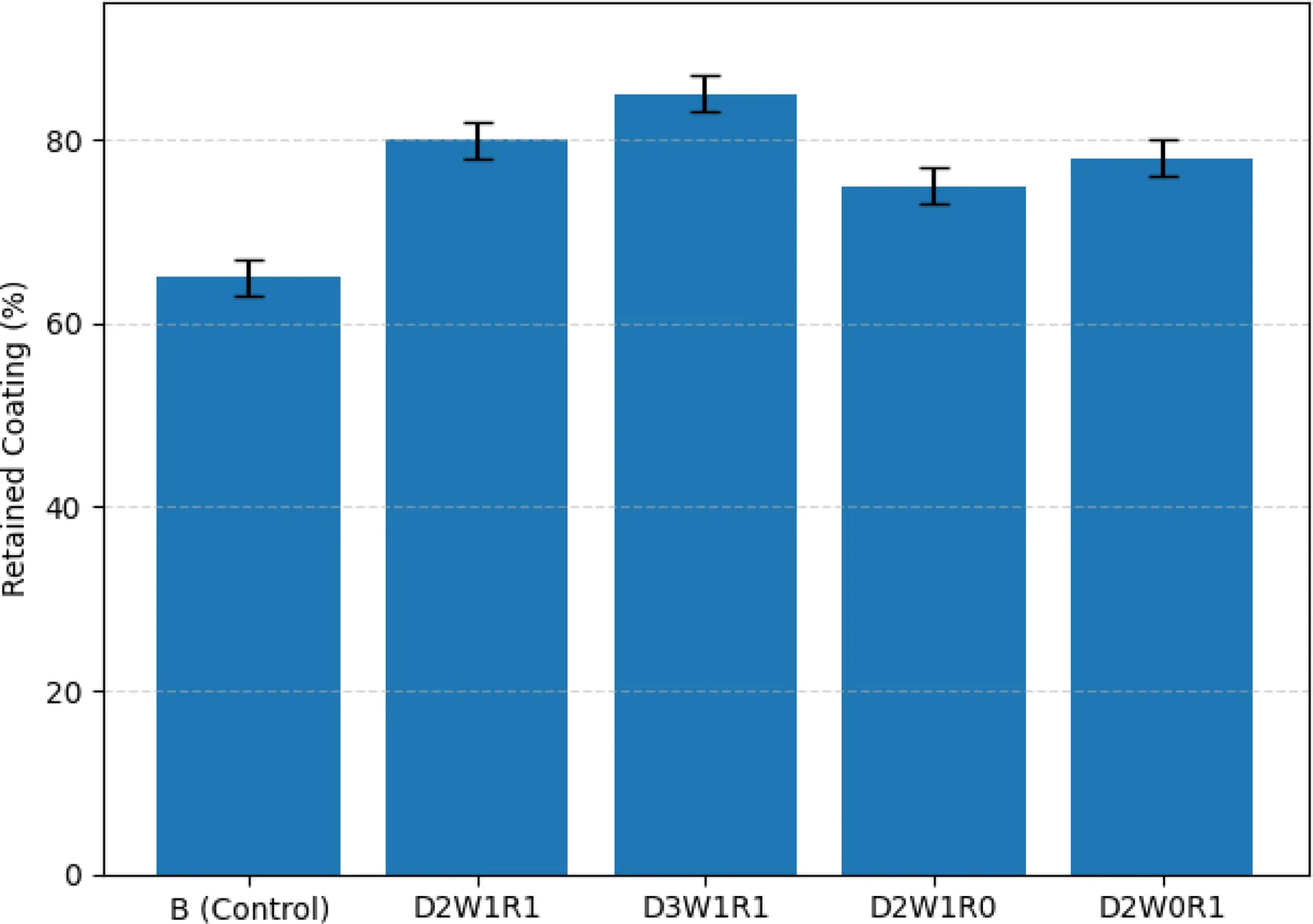
Retained coating ratio of asphalt binders after water boiling test.

Figure 5 illustrates the retained coating ratio of asphalt binders on aggregate surfaces after the water boiling test, which is a qualitative measure of moisture susceptibility and binder aggregate adhesion. A higher retained coating ratio indicates stronger adhesion and better resistance to moisture induced stripping. The control sample (B) exhibited the lowest retained coating ratio (~ 65%), suggesting weaker adhesion under wet conditions. In contrast, modified binders showed substantial improvements. Notably, D3W1R1 achieved the highest retention rate (~ 85%), indicating significantly enhanced moisture resistance. The improved performance can be attributed to the synergistic effects of DFNS, WCO, and WRP. The nano fibrous structure of DFNS may enhance the physical interlocking and mechanical anchorage between the binder and aggregate. Simultaneously, WRP likely contributes through its elastomeric nature, promoting cohesive strength at the interface. While WCO has a plasticizing role, it does not appear to impair adhesion when used in balanced formulations, as evidenced by the relatively high coating ratios in D2W1R1 and D2W0R1 (both around 78–80%). These findings suggest that the DFNS/WCO/WRP modified binders not only improve mechanical and thermal performance, but also strengthen moisture resistance, making them promising candidates for asphalt applications in wet or freeze thaw environments.

### Storage stability

**Fig. 6:**
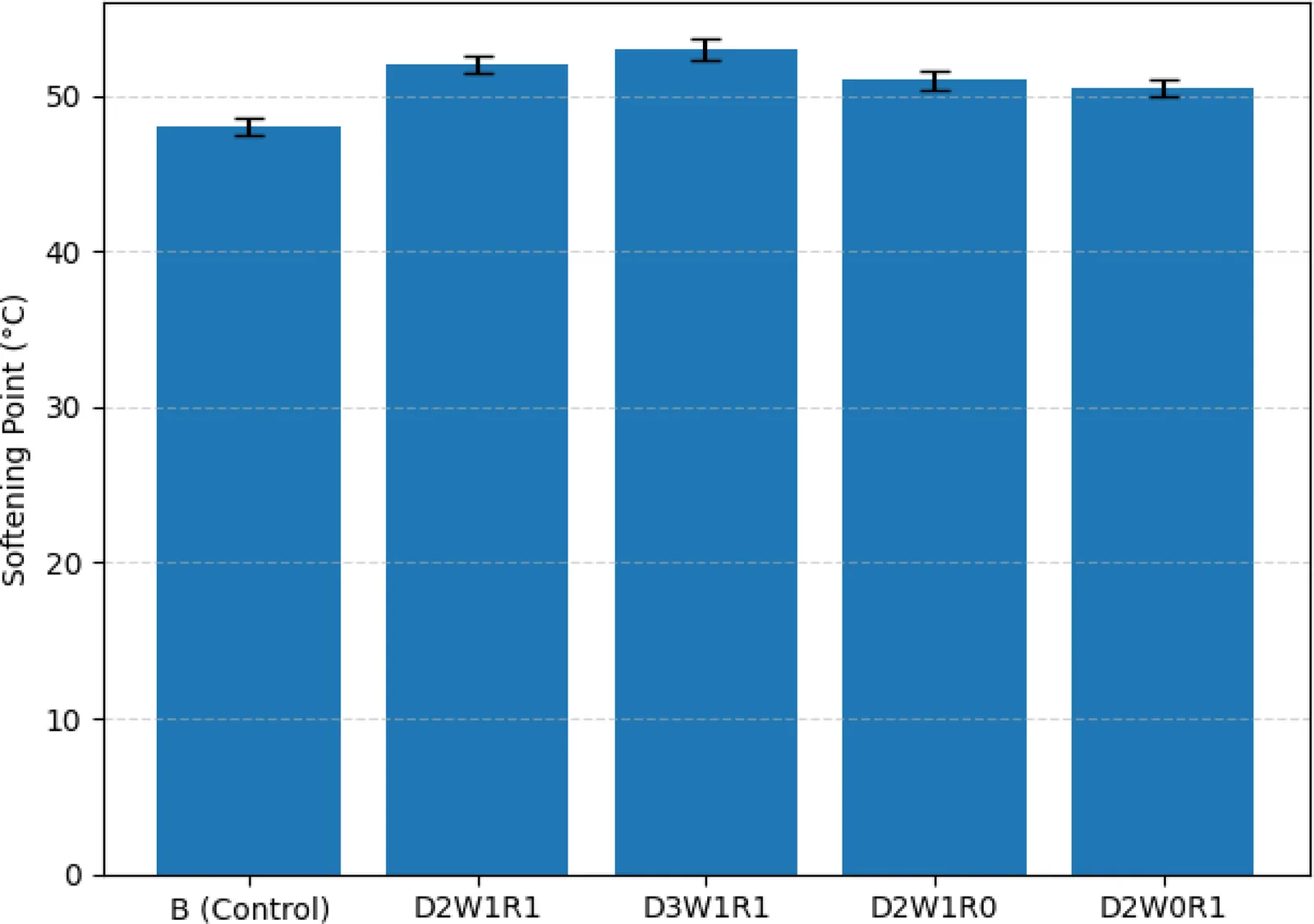
Softening point of top segment of asphalt binders after 48 h storage at 163 °C.

**Fig. 7:**
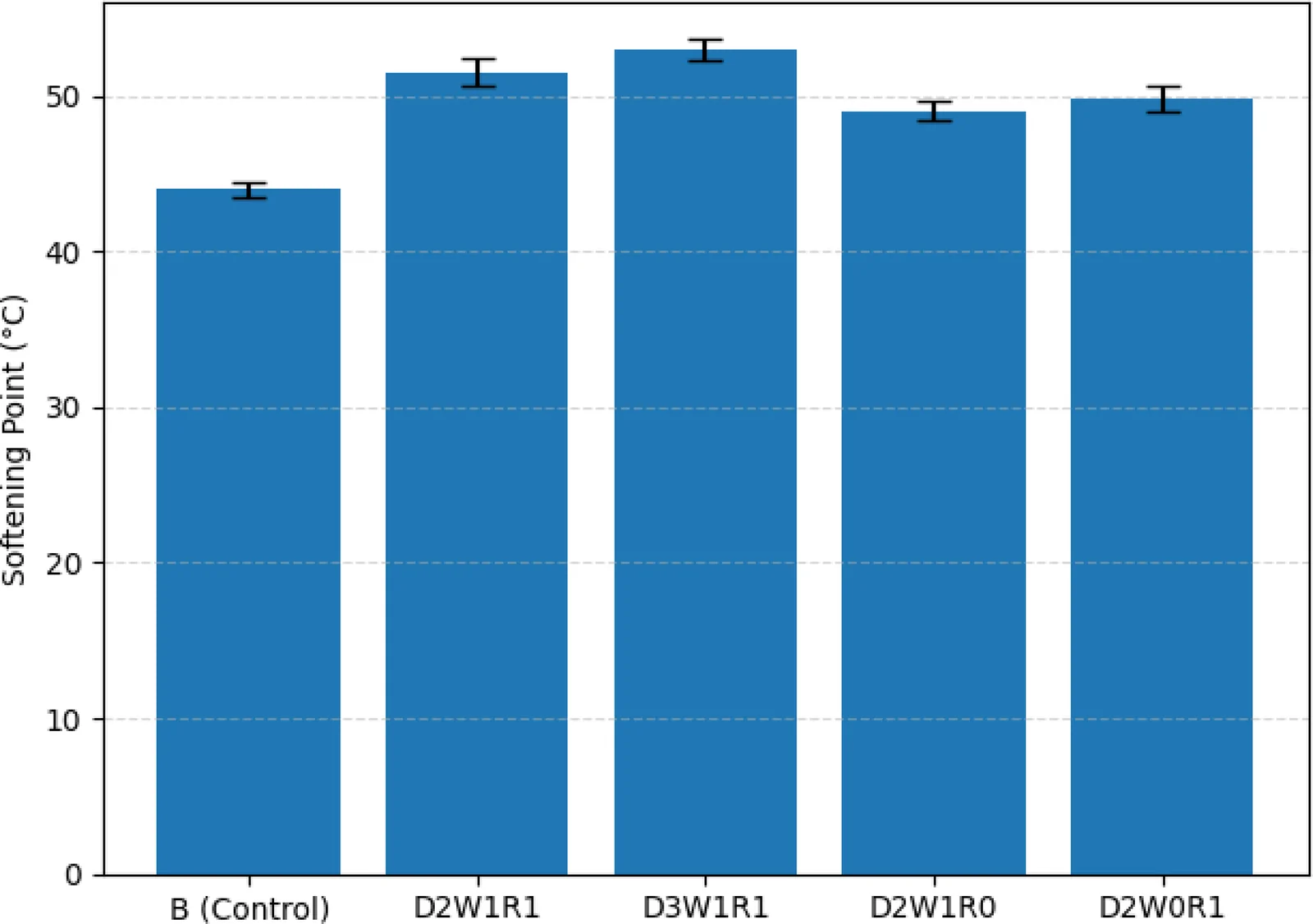
Softening point of bottom segment of asphalt binders after 48 h storage at 163 °C.

**Fig. 8:**
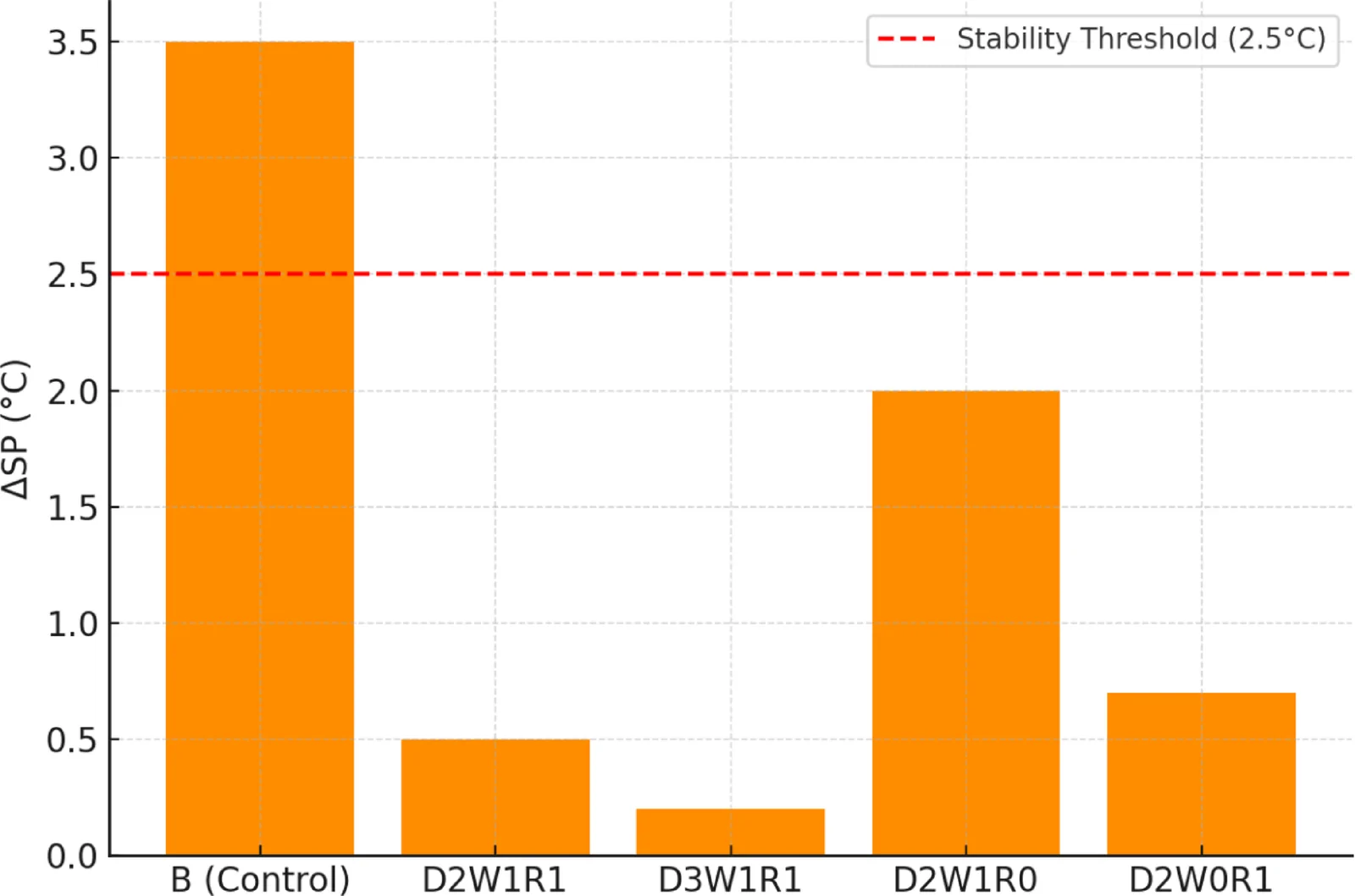
Difference in softening point (ΔSP) between top and bottom segments (Storage Stability Test).

Figures 6, 7 and 8 depict the results of the storage stability test, showcasing the softening point of the top and bottom segments of asphalt binders and the resulting ΔSP (softening point difference). A ΔSP > 2.5 °C is typically considered indicative of phase separation and poor thermal stability. The control sample exhibited a ΔSP of 3.5 °C, clearly exceeding this limit, thus demonstrating pronounced sedimentation or migration of components during storage. It should be noted that the base binder used in this study is a conventional 70# penetration grade asphalt and is not polymer modified. The relatively high softening point difference (ΔSP) observed for the control sample may be attributed to thermal conditioning at elevated temperature (163 °C for 48 h), which can induce redistribution of asphaltene and maltene fractions even in unmodified binders. This behavior has been reported in previous studies and reflects the intrinsic thermal instability of the base asphalt under prolonged heating conditions. Similar improvements in storage stability after incorporation of nanomaterials have been attributed to improved dispersion and stronger interfacial interactions within the asphalt matrix^28,31^. In contrast, the modified binders, particularly D3W1R1 (ΔSP = 0.2 °C) and D2W1R1 (ΔSP = 0.5 °C), maintained highly stable profiles with minimal top bottom discrepancies. These observations reflect the reinforcing effect of DFNS and the compatibilizing nature of WCO, which, like SA in prior studies, likely improves the dispersion of polymeric or particulate modifiers (such as WRP) by reducing surface tension and enhancing interfacial cohesion. The fibrous network of DFNS may also limit vertical mobility of denser components, thereby preserving uniformity. Interestingly, WCO alone (D2W0R1) produced moderate stability (ΔSP ≈ 0.7 °C), confirming its beneficial role. However, formulations lacking either DFNS or WRP, such as D2W1R0, experienced more notable separation (ΔSP = 2.0 °C), emphasizing that synergistic interaction among DFNS, WCO, and WRP is essential for long term thermal homogeneity (Figs. 6, 7 and 8). Overall, composite modification with balanced WCO and structured additives improved the storage stability of asphalt binders by enhancing physical integrity and dispersion behavior, consistent with observations reported for other nanostructured asphalt systems. The base binder used in this study was supplied as a conventional 70# penetration-grade petroleum asphalt and was not modified with polymer additives during sample preparation. The relatively high softening point difference (ΔSP) observed for the control sample may be associated with prolonged thermal conditioning at elevated temperature (163 °C for 48 h), which can promote redistribution of asphaltene and maltene fractions and induce compositional heterogeneity within the binder. Although this behavior is not typically expected for unmodified binders, previous studies have suggested that extended thermal exposure may alter the colloidal balance and internal structure of asphalt systems, potentially resulting in measurable softening point differences after prolonged storage conditioning^38,39^.

### Thermal properties

**Fig. 9:**
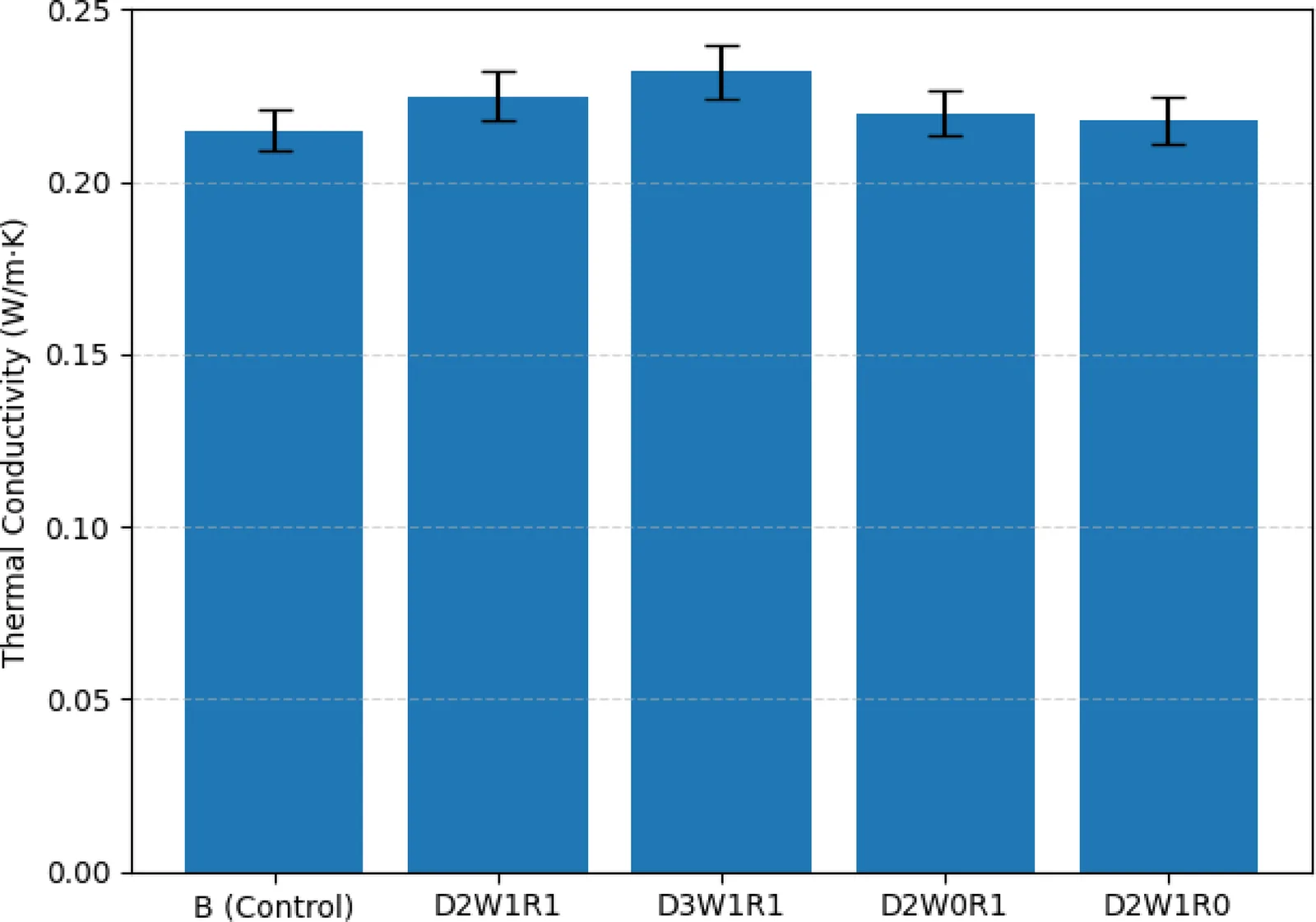
Thermal conductivity (W/m·K) of DFNS/WCO/WRP modified asphalt binders.

Figure 9 shows the thermal conductivity of asphalt binders at 25 °C, providing insight into their ability to dissipate or insulate heat. The control binder (B) demonstrated the lowest thermal conductivity (~ 0.215 W/m·K), whereas the modified samples especially D3W1R1 (~ 0.233 W/m·K) showed noticeable enhancement in heat conduction. This trend contrasts with previous studies on aerogel (SA) based binders, which typically exhibited reduced thermal conductivity due to the three dimensional porous network of SA, known to disrupt and elongate heat transfer pathways. However, in our case, the inclusion of DFNS and WRP, rather than highly porous SA, resulted in better thermal continuity within the asphalt matrix likely due to their denser microstructure and uniform dispersion enabled by WCO. The observed increase in conductivity, particularly in D3W1R1, can be explained by the presence of DFNS as a silica rich nanomaterial that facilitates efficient thermal conduction, unlike aerogels that act as insulators. Unlike highly porous silica aerogels that reduce heat transfer, DFNS in the present system formed a denser and more continuous microstructural network within the asphalt matrix, which may facilitate heat conduction and explain the observed increase in thermal conductivity. Furthermore, WCO appears to assist in the homogeneous distribution of modifiers, minimizing microvoids and enhancing matrix cohesion. Interestingly, in other systems where SA was used, WCO played a dual role: improving dispersion at low SA contents (reducing conductivity) and disrupting porous structure at higher SA contents (increasing conductivity). While our system does not contain aerogel SA, similar interactions between modifier structure and WCO can be inferred highlighting that thermal performance is strongly influenced by modifier morphology, phase distribution, and matrix integrity. Overall, these findings indicate that DFNS/WCO/WRP modified binders enhance thermal conductivity beneficial for applications requiring rapid heat dissipation, such as in hot climates or under high temperature traffic loads. However, for applications requiring thermal insulation, the design of the modifier system must prioritize porosity and low density components.

**Fig. 10:**
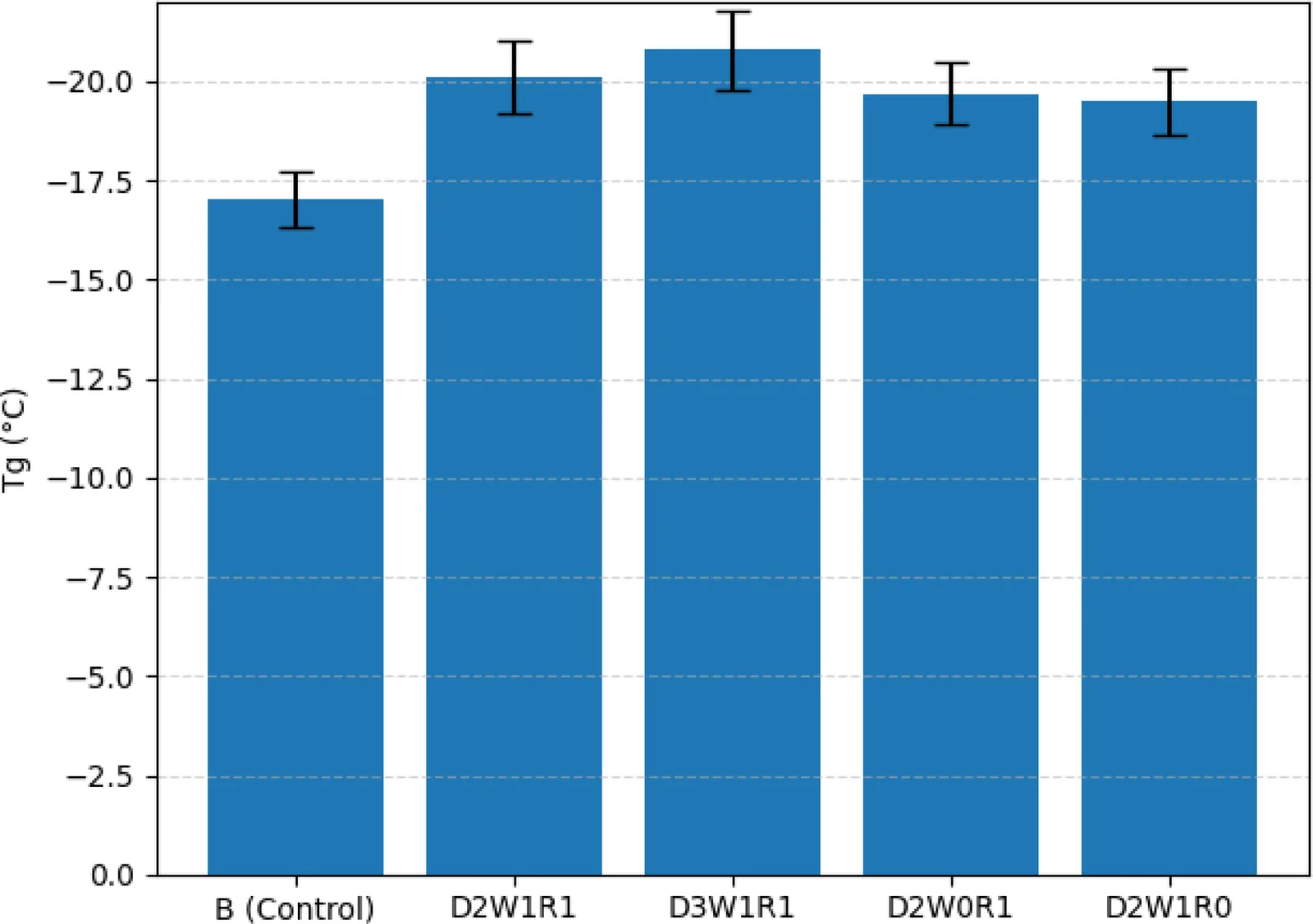
Effect of DFNS, WCO, and WRP on Tg of asphalt binders (DSC Test).

Figure 10 presents the glass transition temperature (Tg) of asphalt binders as determined by DSC analysis. Tg is a critical thermal property that reflects the temperature below which the binder transitions from a viscoelastic to a brittle, glassy state. Lower Tg values generally indicate improved low temperature flexibility and better resistance to thermal cracking. The control binder (B) showed the highest Tg (− 17.0 °C), indicating a more rigid behavior at low temperatures. In contrast, all modified binders exhibited lower Tg values, with the most significant reduction observed in D3W1R1 (− 20.8 °C), followed by D2W1R1 (− 20.1 °C). This shift to lower Tg suggests that the inclusion of WCO and DFNS/WRP contributes to softening the asphalt binder matrix, thereby enhancing its ability to deform without fracturing under cold conditions. The WCO component, being oil rich and compatible with the maltene phase of asphalt, acts as a plasticizer that lowers the Tg. Meanwhile, the presence of WRP and DFNS enhances the elastic and structural network, allowing for increased flexibility without compromising thermal stability. The fact that D2W1R0 and D2W0R1 (which lack either WRP or WCO) have slightly higher Tg values compared to D3W1R1, highlights the importance of a balanced formulation to optimize thermal performance. These results indicate that the composite DFNS/WCO/WRP modified asphalt binders exhibit improved thermal adaptability in cold environments, reducing the risk of thermal cracking by maintaining lower Tg values than the base binder.

**Fig. 11:**
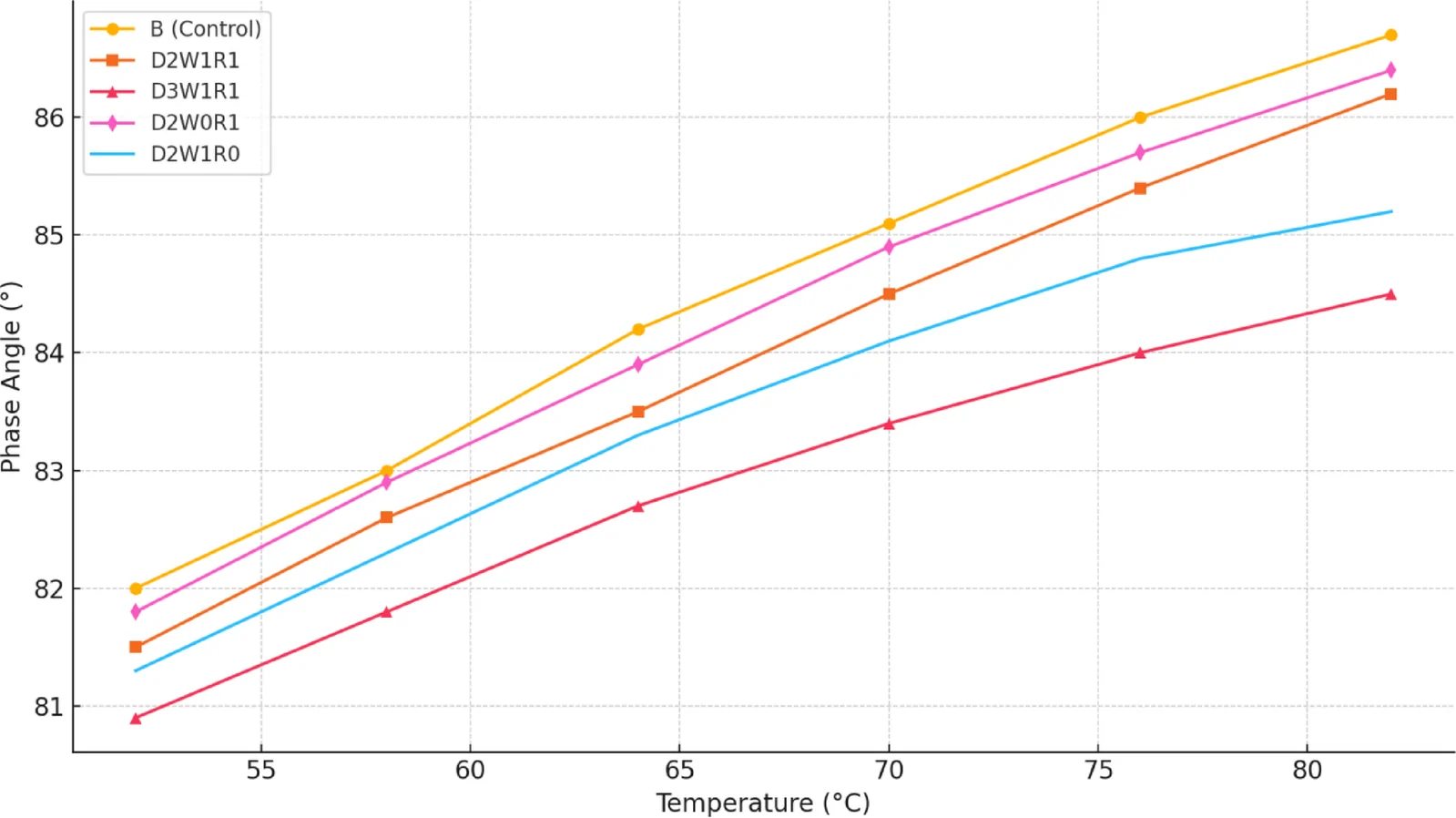
Temperature dependent phase angle behavior of modified asphalt binders under dynamic shear rheometry.

Figure 11 demonstrates the variation of phase angle (δ) of asphalt binders with increasing temperature. Phase angle is a key rheological indicator that reflects the relative dominance of elastic versus viscous behavior. A lower δ implies greater elasticity, while a higher δ indicates more viscous, flow like behavior. In the present study, the DFNS/WCO/WRP modified binders, particularly D3W1R1, exhibited consistently lower phase angles compared to the control binder (B) across the entire temperature range (52–82 °C). This trend suggests that the modified binders have a higher elastic component at elevated temperatures, contributing to better rutting resistance under heavy traffic loads. This behavior parallels findings from prior studies involving SA based modification. There, the formation of a three dimensional internal network within the binder restricted molecular mobility and suppressed viscous deformation. Similarly, in our system, the rigid framework introduced by DFNS and the reinforcing character of WRP appear to limit phase angle growth, maintaining structural coherence even as temperature increases. Moreover, the WCO component, rich in light fractions, typically enhances asphalt fluidity and raises the phase angle. Although WCO generally increases the viscous character of asphalt binders, in the present DFNS/WCO/WRP system this effect was offset by the reinforcing action of DFNS and the elastic contribution of WRP, leading to a net reduction in phase angle. However, in the presence of high DFNS content (e.g., in D3W1R1), this softening effect is overcome by the stiffening influence of the nanofibrous and rubber like modifiers, leading to a more stable viscoelastic response. The increase in complex modulus and the reduction in phase angle can be attributed to the reinforcing framework provided by DFNS and the elastic contribution of WRP, while WCO improves dispersion and prevents excessive stiffening of the binder. In summary, the balanced interaction among DFNS, WCO, and WRP produces a modified binder that maintains lower phase angles and greater elasticity at high temperatures, indicative of superior high temperature performance without sacrificing flexibility.

**Fig. 12:**
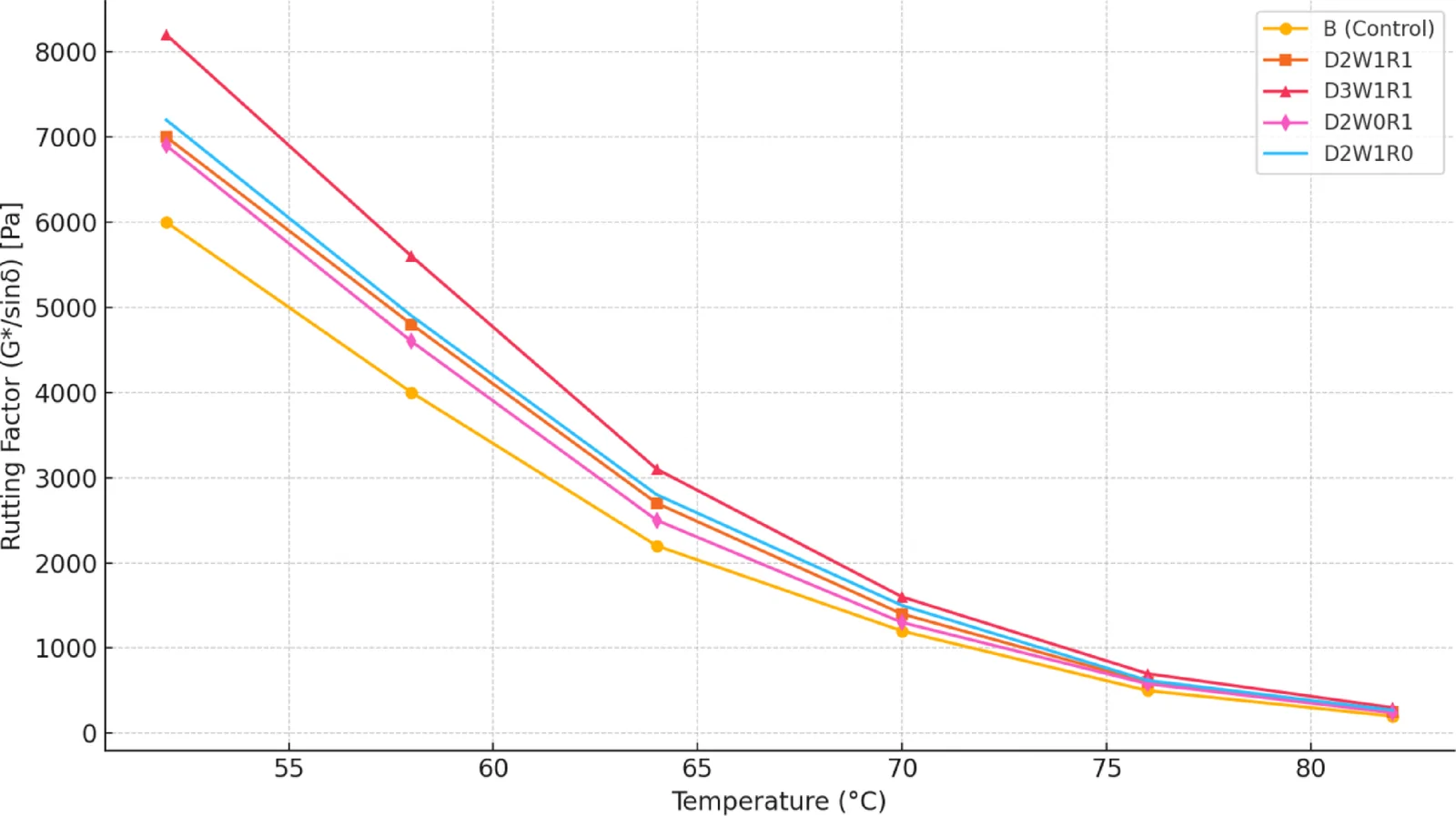
Variation of rutting factor (G*/sinδ) with temperature for DFNS/WCO/WRP modified asphalt binders compared to control binder.

Figure 12 illustrates the variation of the rutting factor (G*/sinδ) of asphalt binders with temperature, a rheological parameter used to evaluate high temperature deformation resistance. Higher G*/sinδ values represent greater stiffness and reduced susceptibility to permanent deformation under traffic loading. In the control sample (B), the rutting factor declined sharply with increasing temperature, indicating poor resistance to rutting at elevated temperatures. In contrast, modified binders, especially D3W1R1, maintained significantly higher G*/sinδ values throughout the temperature range (52–82 °C). This suggests that the incorporation of DFNS and WRP, similar to SA in prior studies, greatly enhances the elastic stiffness of the binder, thereby improving high temperature structural stability. Similar to observations with SA modified systems, the inclusion of WCO in the DFNS/WCO/WRP composite introduced a softening effect, slightly reducing G*/sinδ due to the increased fluidity of the asphalt matrix. However, this effect was largely mitigated when the structural reinforcement from DFNS and WRP was sufficient particularly in the D3W1R1 formulation, which showed the highest rutting resistance. These results highlight the synergistic interaction among the three modifiers: DFNS contributes stiffness and thermal stability, WRP enhances elasticity, and WCO facilitates dispersion but must be balanced to avoid excessive softening. The optimum rutting resistance is achieved through careful tuning of these components.

**Fig. 13:**
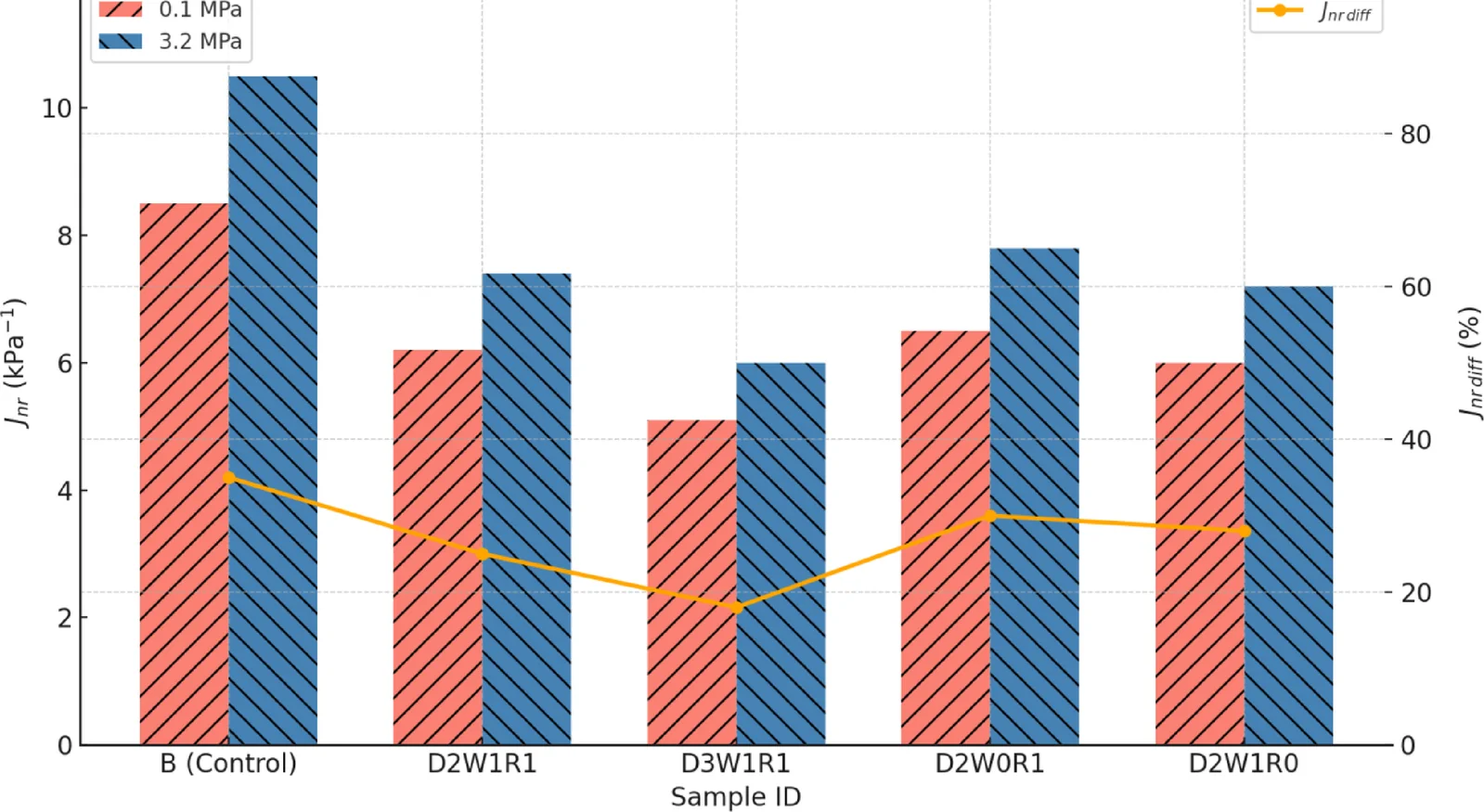
*J*_*nr*_ and *J*_*nrdiff*_ of different types asphalt.

Figure 13 displays the results of the Multiple Stress Creep Recovery (MSCR) test for modified asphalt binders, focusing on two principal parameters: non recoverable creep compliance (*J*_*nr*_) under two stress levels (0.1 MPa and 3.2 MPa), and the corresponding stress sensitivity index (*J*_*nrdiff*_). In the control sample (B), *J*_*nr*_ values are significantly higher under both stress levels, indicating poor resistance to permanent deformation. The incorporation of DFNS and WRP, particularly in D3W1R1, leads to a marked reduction in *J*_*nr*_ values, highlighting enhanced creep resistance and a more stable elastic recovery structure. This improvement is attributed to the rigid nanostructure of DFNS and the elastic reinforcement effect of WRP. The lower Jnr values observed for DFNS/WCO/WRP modified binders suggest that the rigid structure of DFNS and the elastic nature of WRP enhance resistance to permanent deformation, while WCO improves compatibility among the modifiers. On the other hand, samples containing WCO show a moderate increase in J_nr_ values, especially at 2% DFNS content, which implies that WCO rich in light fractions introduces softening behavior and greater susceptibility to permanent strain. However, when combined with a higher content of DFNS (as in D3W1R1), this adverse effect is effectively neutralized. Moreover, *J*_*nrdiff*_, which quantifies stress sensitivity, shows that DFNS can lower sensitivity up to an optimal point. Excessive DFNS content may slightly increase stress sensitivity, possibly due to microstructural rigidity limiting recovery under stress. The combination of WCO with DFNS/WRP, however, appears to balance deformation resistance and stress responsiveness effectively. Overall, the MSCR analysis confirms that carefully tuned DFNS/WCO/WRP formulations, particularly with a higher DFNS ratio, significantly improve the permanent deformation resistance of asphalt binders while keeping stress sensitivity within acceptable limits.

### Microstructural analysis

**Fig. 14:**
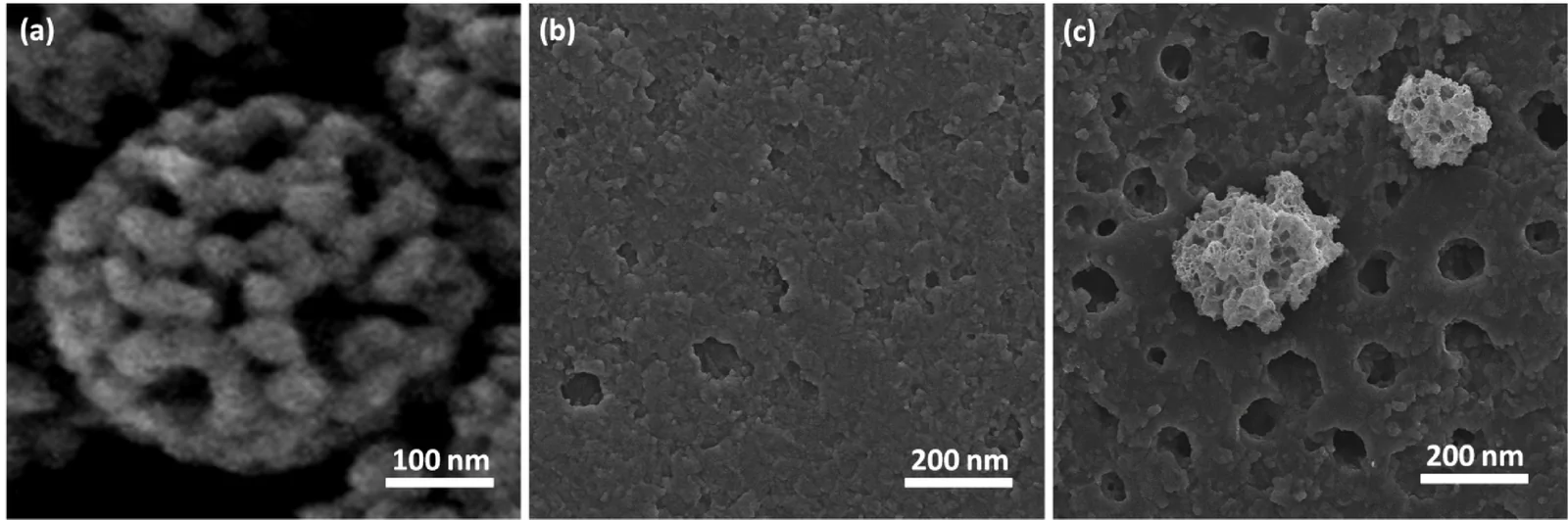
SEM micrographs of asphalt binder surfaces: (**a**) DFNS, (**b**) unmodified binder, (**c**) DFNS modified binder.

The SEM micrographs in Fig. 14 reveal the surface morphology of DFNS. As depicted in Fig. 4a, the DFNS sample exhibits parapet like structures with consistent proportions and dendrimer filaments with thicknesses ranging from 350 to 400 nm, arranged in three dimensions to form the boundaries (Fig. 14a). To further investigate the microstructural interaction between modifiers and asphalt binder, SEM images of DFNS/WCO/WRP modified asphalt samples were analyzed. As shown in the SEM micrographs (Fig. 14b and c), the surface morphology of the modified asphalt reveals a distinct network like structure and notable surface roughness compared to the relatively smooth base asphalt. The observed porous features and embedded spherical or flake like DFNS particles confirm the successful physical incorporation of nanostructured silica into the asphalt matrix. The sample with higher DFNS content (D3W1R1) shows a denser distribution of micro voids and surface undulations, suggesting the formation of a reinforcing three dimensional network, which could be attributed to the high surface area and rigid skeleton of DFNS. These features are indicative of enhanced structural rigidity and improved elastic recovery. However, the presence of micropores ranging from 400 nm, possibly caused by the detachment of DFNS agglomerates or air entrapment during mixing, also highlights the challenges in achieving uniform dispersion and strong interfacial bonding between DFNS and the asphalt medium.

The comparative analysis between conventional spherical SiO₂ nanoparticles and DFNS reveals that DFNS modified binders exhibit superior performance across multiple properties. The G*/sinδ value for DFNS modified asphalt (D1 and D2) is significantly higher than that of SiO₂ samples (S1 and S2), indicating enhanced high temperature rutting resistance. Moreover, BBR results demonstrate that DFNS leads to a lower stiffness modulus and higher m-value at low temperatures, evidencing improved flexibility and crack resistance. The superior performance of DFNS is attributed to its dendritic, high surface area fibrous structure, which allows for better dispersion and stronger physical interaction with the asphalt matrix compared to compact spherical SiO₂ NPs. This structure also contributes to the observed reduction in thermal conductivity, enhancing insulation performance. Furthermore, SEM images confirm that DFNS creates a more interconnected microstructure, while spherical SiO₂ exhibits limited integration. These results collectively demonstrate that DFNS is a more effective nanomaterial for multifunctional asphalt enhancement compared to traditional SiO₂ NPs (Table 10).

**Table 10:** Comparative evaluation of rheological and thermal properties of asphalt modified with DFNS and spherical SiO₂ nanoparticles.

Sample ID	Explanation	Viscosity (Pa·s)	G*/sinδ (kPa)	S (MPa)	m-value	Thermal conductivity
B	Base Asphalt (control)	0.46	1.05	0.46	0.295	0.260
S1	SiO₂ NPs (3%)	0.54	1.38	0.54	0.310	0.240
D1	DFNS (3%)	0.59	1.65	0.59	0.328	0.210
S2	SiO₂ NPs (3%) + WCO (3%)	0.51	1.31	0.51	0.315	0.228
D2	DFNS (3%) + WCO (3%)	0.56	1.60	0.56	0.335	0.195

### Mechanism of performance enhancement in DFNS/WCO/WRP modified asphalt

The improved performance of the DFNS/WCO/WRP modified asphalt binder can be attributed to the complementary roles of the three modifiers. DFNS, with its dendritic fibrous structure and high surface area, forms a reinforcing microstructural framework within the asphalt matrix that enhances stiffness, rutting resistance, and structural stability. Waste cooking oil (WCO) acts primarily as a rejuvenating agent, restoring the light fractions of asphalt and improving flexibility and low temperature performance. Meanwhile, waste rubber powder (WRP) contributes elastomeric behavior, improving elasticity, fatigue resistance, and durability under repeated loading. When used together, these modifiers create a synergistic system in which WCO improves dispersion and compatibility, DFNS provides structural reinforcement, and WRP introduces elastic recovery. This combined effect results in balanced improvements in conventional properties, rheological behavior, thermal stability, and fatigue performance of the asphalt binder.

## Conclusions

This study evaluated the effects of DFNS, WCO, and WRP on the physicochemical, rheological, thermal, and microstructural properties of asphalt binders. The results demonstrated that the combined incorporation of these modifiers significantly enhanced deformation resistance, fatigue performance, and overall viscoelastic behavior. DFNS improved stiffness and rutting resistance due to its fibrous nanostructure and high surface area, while WCO acted as a rejuvenator, restoring light fractions and enhancing low temperature flexibility. WRP contributed elastomeric behavior, improving elastic recovery and resistance to fatigue under cyclic loading. The synergistic interaction among these components resulted in a balanced improvement in both high and low temperature performance. Rheological tests (DSR, BBR, MSCR, and time sweep) confirmed enhanced viscoelastic properties and resistance to permanent deformation. SEM analysis indicated improved dispersion and the formation of a more uniform internal structure, while thermal analysis showed a slight increase in thermal conductivity with DFNS incorporation. Statistical analysis using RSM revealed that WRP had the most significant influence on conventional performance indicators. Overall, the DFNS/WCO/WRP system offers a promising and sustainable approach for improving asphalt binder performance. However, further studies are recommended to evaluate mixture level behavior, long term aging, and field performance under real conditions.

## Data Availability

All data generated or analysed during this study are included in this published article.

## Abbreviations

DFNS: Dendritic fibrous nanosilica
WCO: Waste cooking oil
WRP: Waste rubber powder
NPs: Nanoparticles
SEM: Scanning electron microscope
XRD: X-ray diffraction
FTIR: Fourier transform infrared spectroscopy
BET: Brunauer-Emmett-Teller
CPB: Cetylpyridinium bromide
AFM: Atomic force microscopy
TEM: Transmission electron microscopy
DBJH: Average pore diameter
TEOS: Tetraethyl orthosilicate
SA: Silica aerogel
RSM: Response surface methodology
